# Three-dimensional observation and analysis of remineralization in dentinal caries lesions

**DOI:** 10.1038/s41598-020-61111-1

**Published:** 2020-03-09

**Authors:** Kumiko Yoshihara, Noriyuki Nagaoka, Akiko Nakamura, Toru Hara, Satoshi Hayakawa, Yasuhiro Yoshida, Bart Van Meerbeek

**Affiliations:** 1 National Institute of Advanced Industrial Science and Technology (AIST), Health Research Institute, 2217-14 Hayashi-Cho, Takamaysu, Kagawa 761-0395 Japan; 2 Okayama University, Graduate School of Medicine, Dentistry and Pharmaceutical Sciences, Department of Pathology & Experimental Medicine, 2-5-1 Shikata-cho, Kita-ku, Okayama, 700-8558 Japan; 3 Okayama University Dental School, Advanced Research Center for Oral and Craniofacial Sciences, 2-5-1 Shikata-cho, Kita-ku, Okayama 700-8558 Japan; 4 National Institute for Materials Science, 1-2-1 Sengen, Tsukuba, Ibaraki 305-0047 Japan; 5 Okayama University, Graduate School of Interdisciplinary Science and Engineering in Health Systems, Biomaterials Laboratory, 3-1-1, Tsushima-naka, Kita-ku, Okayama 700-8530 Japan; 6 Hokkaido University, Faculty of Dental Medicine, Department of Biomaterials and Bioengineering, Kita 13, Nishi 7, Kita-ku, Sapporo, Hokkaido 060-8586 Japan; 7 KU Leuven (University of Leuven), Department of Oral Health Research, BIOMAT & UZ Leuven (University Hospitals Leuven), Dentistry, Kapucijnenvoer 7, 3000 Leuven, Belgium

**Keywords:** Scanning electron microscopy, Dental caries

## Abstract

The remineralization mechanism in dental caries lesions is not completely understood. This study reports on ultrastructural and chemical changes observed within arrested caries lesions. Carious human teeth were observed using scanning electron microscopy (SEM) and focused-ion-beam (FIB)-SEM. The crystals detected in the caries lesions were characterized by transmission electron microscopy (TEM), along with chemical element mapping using energy-dispersive spectroscopy (EDS)-STEM. FIB-SEM 3D reconstructions revealed a severely damaged dentin surface abundantly covered by bacteria. Although the dentin tubules were clogged up to a depth of 100 μm, bacterial invasion into dentin tubules was not observed. TEM crystal analysis and EDS-STEM revealed the presence of Ca and P, as well as of Mg within the HAp crystals deposited inside the dentin tubules. It was concluded that extensive remineralization with deposition of Mg-HAp crystals had occurred in dentin tubules of caries-arrested dentin. Understanding the natural remineralization process is thought to be helpful for developing clinical biomimetic remineralization protocols.

## Introduction

Dental caries is the most prevalent health condition in the world^1,2^. This disease is a dysbiosis caused by undisturbed bacterial accumulations, which can be up regulated by the presence of fermentable carbohydrates and down regulated by fluoride. Although salivary flow rate has its role in the process, the general and main etiologic cause is undoubtedly undisturbed biofilm accumulations with release of metabolic acids leading to substantial mineral loss from the tooth structure^3,4^. When the pH of the oral cavity is neutral and saliva is sufficiently saturated with calcium and phosphate, remineralization by mineral deposition will occur^5^. The caries process is well documented to progress when the balance between demineralization and remineralization is disturbed^6^. However, the process of caries initiation is not yet fully described and understood.

Remineralization of carious dentin has been investigated before. Lesters and Boyde^6^ documented the crystal formation and described the crystal structure of enamel and dentinal caries using SEM and TEM. Ogawa *et al*. investigated the transparent and sub-transparent layers of caries lesions by SEM and measured the different layer’s surface hardness. They found that apatite was deposited in the tubules of the transparent and sub-transparent caries layers^7^. Frank and Voegel^8^ also observed partially and completely calcified dentin tubules in the area between the translucent zone of carious dentin and inner dentin. However, they did not report the presence of remineralized dentin in the immediate surrounding of the carious lesion. Later, several researchers documented the crystal structure of demineralized dentin using TEM^8–11^. Zavgorodny *et al*.^10,11^ observed a transparent zone close to the demineralized caries area. This transparent zone constitutes carious dentinal tissue with intratubular mineralization. In their study, remineralization occurred only partially and did not completely clog the dentinal tubules. Although many of these researches involved nano-scale observations of remineralization, the 3D structural organization of naturally remineralized dentin was not completely disclosed.

Counteracting caries development, active bio-remineralization using calcium phosphate compounds or bioactive glasses has thoroughly been studied^12^. Casein phosphate is for instance known to inhibit demineralization of enamel *in vitro*; clinical evidence of remineralization of enamel and dentin by casein phosphate is however today still lacking^13^. Likewise, bioactive glass was shown to have remineralization potential *in vitro*, while this effect has so far not been proven *in vivo*^14^. Another remineralization strategy concerns biomimetic analogs of noncollagenous proteins^2,15^. Investigating remineralization, most studies have employed SEM and TEM to study *in vitro* remineralization in 2D, while the actual 3D process of natural *in vivo* remineralization has not been completely described thus far. Hence, an in-depth *in/ex vivo* study of caries and the involved de- and remineralization processes in human teeth may be very helpful to develop clinical strategies to achieve efficient tooth remineralization.

To better understand the lateral extent and thickness of remineralization, 3D Focused-ion-beam Scanning Electron Microscopy (FIB-SEM) imaging was conducted in this study. FIB-SEM is a research technique enabling to study the structure of 3D volumes by wide-area high-resolution 3D reconstruction of consecutively taken SEM photomicrographs. In the biological field, FIB-SEM has for instance been applied to image nano-structures of cells such as at the tendon-bone interface^16^ and periodontal ligament^17^, and also to observe specific tooth-related aspects such as dentin cracks^18^ and adhesive-tooth interfaces^19^. In addition, FIB-SEM is a useful tool for preparing TEM specimens of precisely selected areas^20^. TEM can provide high-resolution structural information along with site-specific chemical analytic data when combined with Energy-Dispersive X-ray Spectrometry (EDS), as is commonly used in scanning TEM or STEM mode, and with crystallographic information when combined with Selected Area Electron Diffraction (SAED). The primary purpose of this exploratory study was to characterize actual *in-vivo* remineralization within dentinal caries, correlatively utilizing structural FIB-SEM imaging with EDS-STEM chemical and SAED crystal analysis.

## Results

**Figure 1:**
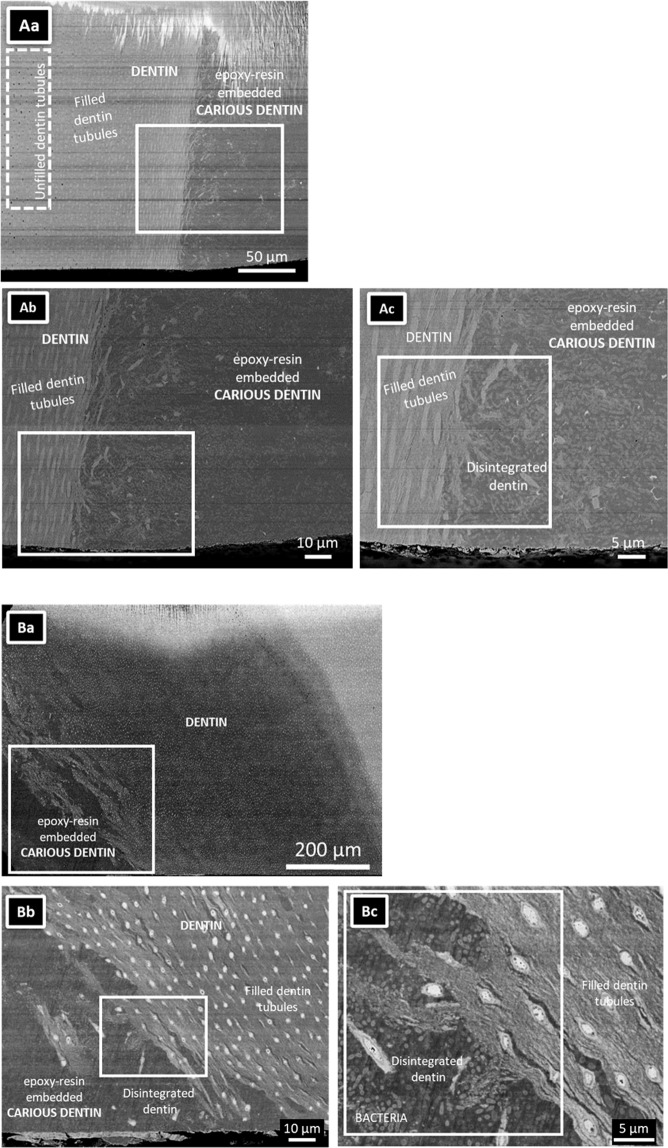
SEM photomicrographs of a single section through two arrested caries lesions originating from two different carious teeth. **(Aa,Ba)** Overview SEM photomicrographs of the dentinal caries lesions, illustrating that the caries lesion extended into deep dentin exhibiting many parallel running dentin tubules. The specimens were embedded in epoxy resin. The dotted line demarcates the transition of the caries lesion to the adjacent caries-affected dentin. Note that the specimen top part was damaged by the argon-ion beam of the cross-section polisher (JEOL). **(Ab,c)** High-magnification SEM photomicrographs revealed open dentin tubules relatively far away from the dentinal caries lesion. Clearly filled dentin tubules were observed near the interface (up to about 100 μm remote from the interface). **(Ac)** High-magnification photomicrographs confirming that the dentin tubules near the interface with the caries lesion were filled with highly dense material. Collapsed dentin with bacteria was observed near the dentin-caries interface. **(Ba)** A deep caries region was observed on the lower left of the image. **(Bb)** High-magnification of (**Ba**). Collapsed collagen was observed at the interface of the caries lesion with dentin. Filled dentin tubules were observed. **(Bc)** High-magnification SEM imaging revealing the presence of a considerable number of bacteria at the caries lesion.

Low-magnification SEM disclosed an invasive dentinal caries lesion (Fig. 1Aa–c). Open dentin tubules were observed in dentin far away from the carious tissue (left side of the caries lesion), while filled tubules were observed near the caries lesion (Fig. 1Aa). Higher-magnification SEM revealed a destroyed dentin structure at the caries lesion. Disintegrated dentin along with a considerable number of bacteria were embedded in epoxy resin (Fig. 1Ab). The dentin tubules near the caries lesion were filled with a high-density substance (Fig. 1Ac). SEM of the arrested caries lesion in the second tooth revealed a similar structure (Fig. 1Ba–c). Severely damaged dentin contained voids and bacteria, while the dentinal tubules were filled with a dense substance (Fig. 1Bc).

**Figure 2:**
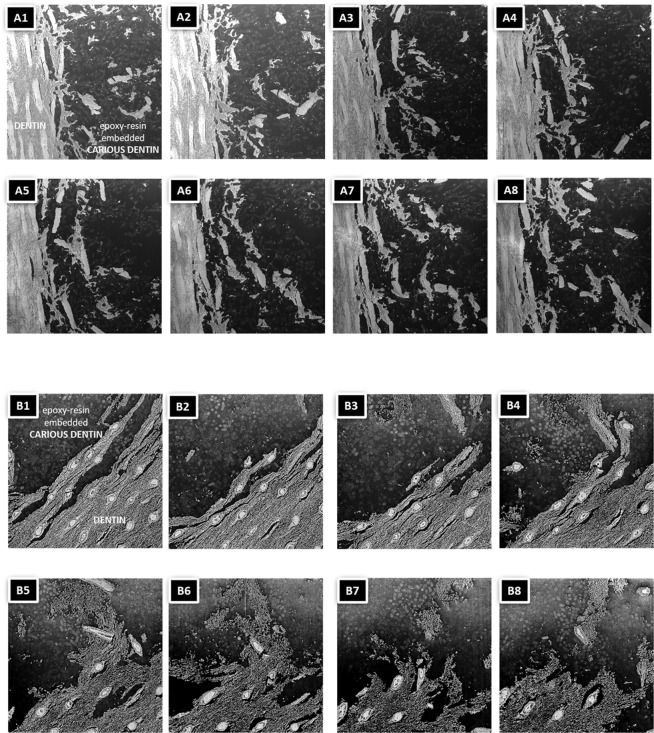
(**A,B**) SEM photomicrographs of two FIB-SEM section series of 30-nm thick slices of the squares marked in Fig. 1Ad,Bc, respectively. Slices number at 25 (a/b1), 175 (a/b2), 325 (a/b3), 475 (a/b4), 625 (a/b5), 775 (a/b6), 925(a/b7) and 992 (a/b8) are shown. The corresponding 3D FIB-SEM reconstructions are shown in the Extended Figs. 1 and 2, respectively.

3D structural information was obtained by FIB-SEM, showing that the dentinal tissue was severely damaged by caries (Fig. 2A1–8,B1–8). The white substance within the dentin tubules formed a rigid structure and extended clearly within each dentin tubule. At the interface of dentin with the caries lesion, the dentinal tubule structure was fragmented, while the white tubule-filling substance remained nearly intact. Bacteria were observed within dentin but did not penetrate into the dentin tubules (Supplemental Figure).

**Figure 3:**
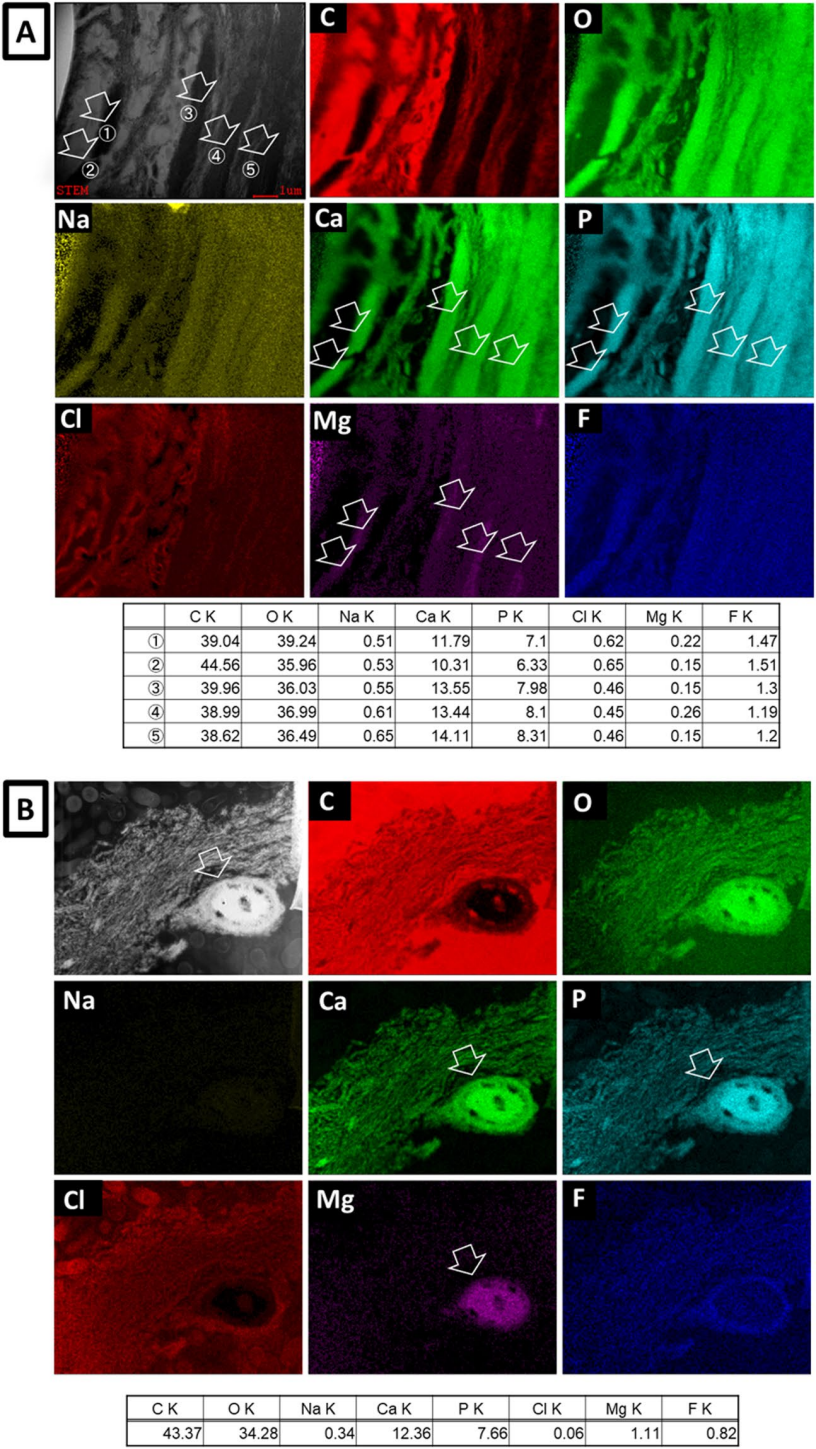
(**A,B**) STEM bright-field image and EDS-STEM mapping of cross-sections through the two arrested caries lesions for carbon (C), oxygen (O), sodium (Na), calcium (Ca), phosphate (P), chlorine (Cl), magnesium (Mg), and fluoride (F). The ion composition (atom%) at each spot (arrows ①–⑤ in 3(**A**) and the arrow in (**B**) for the different chemical elements recorded are summarized in the respective table underneath each mapping.

EDS-STEM elemental mapping at high magnification revealed Ca, O, P and Mg within the highly intratubular substance (Fig. 3A,B: arrows); this substance appeared much denser than intratubular dentin. The Ca/P ratio at sites 1 to 5 in Fig. 3A (arrows) was 1.70, 1.66, 1.70, 1.66 and 1.63, respectively, and that in Fig. 3B (arrow) was 1.61.

**Figure 4:**
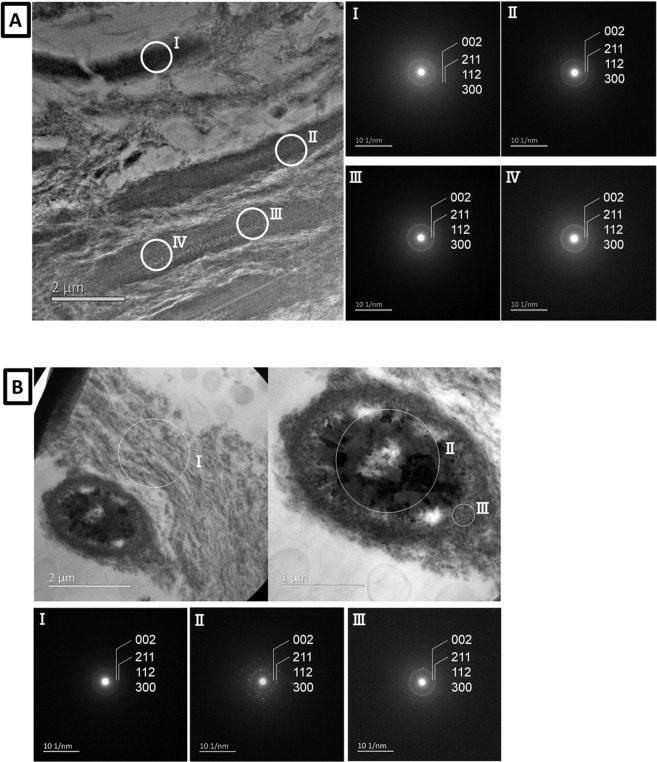
(**A,B**) TEM photomicrograph of a single ultrathin section along with the corresponding electron diffraction pattern (SAED) at each spot ‘I’ to ‘IV’ in (**A**) and ‘I’ to ‘lll’ in (**B**).

This difference in density was confirmed by TEM (Fig. 4A,B). SAED confirmed the presence of hydroxyapatite (HAp) for all spots marked (Fig. 4Aa–d,Ba–c)^21–23^. TEM confirmed the high density of the intratubular substance in contrast to the low density of dentin. SAED confirmed the presence of HAp in both the intertubular dentin remnants and the intratubular substance^21–23^.

## Discussion

This study investigated the process of caries arrest due to remineralization within caries lesions by correlative ultra-structural characterization using SEM, FIB-SEM and (S)TEM, combined with chemical analysis using EDS-STEM and SAED. First, we observed relatively large areas using SEM, upon which we defined the area for FIB-SEM 3D structural analysis using a serial sectioning method^20^. Finally, we prepared TEM specimens for crystal identification, also using FIB-SEM. FIB-SEM is often used for preparing TEM specimens, because areas with a spatial accuracy of ~10 nm can be selected^20^. In conventional FIB-SEM, the SEM and FIB optical axes intersect one another at ~60°. When imaging serial-sectioned slice planes, proper alignment is crucial to reconstruct the specimen volume in 3D. Using orthogonal FIB-SEM, the incident secondary electron beam is always perpendicular to the FIB-sliced plane. Hence, the contrast of the base plane becomes uniform and the entire dynamic range of the contrast can be used to observe the structure itself, thereby enabling imaging with extremely high contrast^24^.

All teeth exhibiting arrested caries lesions revealed severely destroyed dentin. Despite this dentin destruction, the dentinal tubules near the interface with the caries lesion were filled with a highly dense substance (Fig. 1). Several researchers observed crystal-filled dentin tubules in a vertical cross-section of the tubules^25,26^. 3D FIB-SEM reconstructions in this study clearly revealed precipitate growth in the dentin tubules, even when the surrounding dentin was severely destroyed by caries. Moreover, these precipitate clusters kept their structure, even when separated from dentin due to the caries progression. Surprisingly, bacteria did not penetrate into the dentin tubules, most likely because the tubules were filled at the surface with precipitates, this even though abundant bacteria were present in the dentinal caries. This finding agrees with the observation by Daculsi *et al*.^9^. The progression of an arrested dentin caries has been reported to be slow^26,27^. This slow progression is probably related to the fact that bacteria are not able to infiltrate the dentin tubules. The hardness of chronic/arrested caries is known to be larger than that of acute/active caries, although the hardness of chronic/arrested caries remains below that of sound dentin^28^. This high hardness of chronic/arrested carious dentin is most likely caused by partial remineralization and crystal formation in the carious dentin.

Nucleation and growth of calcium phosphate are important to induce remineralization, which depends on the supersaturation and ionic strength of saliva or dentinal fluid. We could not observe caries lesions and remineralization over time, so we cannot show direct mechanistic evidence of the remineralization process. Nevertheless, two possible mechanisms of intratubular precipitate/crystal formation can be proposed. The first possible mechanism is that remineralization is produced by the odontoblast process or a physicochemical process activated by odontoblast-bacterial interaction^9^. Frank and Voegel have demonstrated remineralization around odontoblast processes^8^. Essential for this mechanism is the presence of the odontoblast process in the dentin tubule. Different studies have discussed the length of the odontoblast process within dentin tubules. Some researchers have reported that odontoblast processes solely remained within the inner side of dentin, less than 1 mm from the pulp^27^, while other researchers have reported that the odontoblast process extends to the outer layer of dentin, even up to the dentin-enamel junction^29^. In our study, odontoblast processes were not observed in the neighborhood of the precipitates. However, it is not known if odontoblast processes are solely effective in their immediate environment with which they make contact or if they can also induce effects at some distance. Daculsi’s proposal also mentioned the additional contributing effect of bacteria; surrounding bacteria may influence remineralization.

A second possible mechanism concerns apatite present in peritubular dentin. Peritubular dentin is highly mineralized^30^. In the current study, carious dentin was partially demineralized with apatite remaining in dentin, especially at peritubular dentin. This residual mineral may serve as nucleus, on which calcium-phosphate crystals grow within the dentin tubules. Hayakawa *et al*.^31^ proposed a mechanism of apatite nucleation and growth, based on an experiment conducted with dry titanium oxide in a narrow-confined space filled with Kokubo’s simulated body fluid (SBF), so to confine the composing ions of apatite^32^. Since precursors of embryos and embryos of apatite remained close to the titanium oxide surface in the confined space, they were thought to contribute to the nucleation and growth of apatite crystallites while consuming Ca and P ions from SBF. Relating these observations to our study, a dentin tubule provides a narrow space and peritubular dentin can act as a nucleation site. Dentinal fluid contains Ca and P ions and flows in the dentin tubules. Therefore, our observation supports the mechanism that the nucleation and growth of calcium phosphate crystals can be initiated at peritubular dentin in the dentin tubules.

To identify the crystal structure of the intratubular precipitates, EDS elemental mapping and SAED were performed. EDS-STEM element mapping revealed a high Ca and P content within the dentinal tubules. In addition, also Na and Mg were detected within the precipitates (Fig. 3). SAED crystal analysis identified the presence of HAp in the intratubular precipitate (Fig. 4), but Mg-ß-TCP was not found despite Mg was detected by EDS-STEM. In literature, the crystals formed during remineralization in dentinal caries have been categorized as Mg-containing ß-tricalcium phosphate (Mg-β-TCP or whitlockite) and Mg-containing HAp (Mg-HAp). Daculsi *et al*. have reported whitlockite and apatite in dentin^26^. At the early stage of remineralization, Mg-ß-TCP (whitlockite) and Mg-poor apatite are formed. Later Mg-ß-TCP is partially converted into Mg-poor apatite. Zavgordniy *et al*.^10,11^ also analyzed the crystals in dentinal caries in two different studies. They first reported the presence of Mg-β-TCP in the carious transparent zone^10^, but later only mentioned the presence of HAp^11^. In our observation, the Ca/P ratio of the intratubular participates varied around 1.6 to 1.7 (Fig. 3). This measured Ca/P ratio supports the formation of HAp^10,33^.

Several specific ions influence the process of remineralization and the actual crystal formation and which type of crystal is formed^34^. Not only our data but also previous studies have revealed the presence of Mg in precipitates or crystals^35^. Abbona *et al*.^36^ reported that HAp can be formed in a low Mg-containing solution (Mg/Ca ratio in solution below 0.4). A higher amount of Mg, such as 0.4 < Mg/Ca in solution < 4.0, forms stable amorphous calcium phosphate or Mg-ß-TCP (whitlockite). On the other hand, HAp is generally formed in SBF solution with a Mg/Ca ratio of 0.6 and pH of 7.4^32^. Mg is typically found in 0.72, 0.44, 1.23 wt% in bone, enamel and dentin, respectively^37,38^; saliva contains about 0.345 mmol/l Mg. The concentration of Mg and Ca ions in saliva depends on the human and physical conditions^39^. EDS revealed a Mg/Ca ratio varying from 0.01 to 0.08 within the intratubular precipitates in our study. The Mg/Ca ratio of Mg-whitlockite or Ca_18_Mg_2_H_2_(PO_4_)_14_ is 0.11^40^. Hence, the Mg concentration detected in the intratubular precipitates must have been too low to form Mg-whitlockite and may therefore have resulted into HAp formation in our study. In this respect, the Mg-whitlockite observed by Daculsi *et al*.^9^ must have been produced in a high Mg-concentrated medium. On the other hand, Zavgorodniy *et al*.^10^ proposed the effect of Si. Silicon is known as an essential element for bone remineralization; Si was shown to induce precipitation of HAp in bone^34^. Silicon may also influence the type of crystal formed during remineralization. Zavgordniy *et al*.^10^ in fact detected Mg, Si and sometimes Cl in addition to Ca and P in the remineralization crystals. The Ca/P ratio of remineralization spots they analyzed was 1.6 and 1.25. The 1.25 Ca/P ratio was much lower than the Ca/P ratio of pure HAp. They explained that Si and Cl may have been inserted into the crystals and that Si may have substituted Mg to form Si-substituted Mg-β-TCP crystals in absence of sufficient Mg. In our observation, Si was not detected. Differences in presence and concentrations of specific ions may influence remineralization and which crystal type is formed in different regions of the caries lesion. In our study, the conditions in the immediate environment around the intratubular precipitates within the arrested caries lesions may have been suitable for HAp formation but not for whitlockite formation.

Meanwhile, Mg is known to inhibit HAp-crystal growth^35,39^. SAED confirmed HAp formation, but TEM revealed that the crystal size of the precipitates in the dentin tubules was less than 100 nm. We cannot deny the co-existence of amorphous calcium phosphates in addition to HAp crystals in the intratubular precipitates. Magnesium may possible be present in both the crystal and amorphous calcium phosphates^10,35^. Magnesium in saliva and Mg released from tooth tissue may have inhibited crystal growth of HAp and formed amorphous carbonate apatite instead. At a later stage, amorphous calcium phosphate can convert to HAp^41^. However, Mg ions adsorbed on the surface of amorphous calcium phosphate retard the transformation to HAp^35^. In the caries-remineralization process investigated in this study, we could not observe the early stage of precipitation. 3D imaging by FIB-SEM indicated that the precipitates formed a tight cluster of HAp crystals and amorphous calcium phosphate, and clogged the dentin tubules. Based on this observation, we suggest that small HAp crystals precipitated and that amorphous calcium phosphate was formed in the dentin tubules due to the salivary environment where Mg exists. The intratubular precipitation may grow with some transformation into additional HAp crystals from the amorphous calcium phosphates. Many *in vitro* studies reported on crystal formation in Mg-containing solutions^35,38,41^. On the other hand, in the mouth several ions exist in saliva and are provided with drinks. When dentinal HAp is dissolved by caries, in addition to several ions, proteins are released and will also influence the remineralization process. It is difficult to observe crystal formation and its growth *in vivo*, although crystal growth is a key element in the process of tooth remineralization. In-depth investigation of the mechanism of nuclei formation and precipitates’ growth in dental caries or the oral environment is needed to understand *in vivo* remineralization.

Furthermore, F has often been reported to play a role in remineralization^12^. Fluoride is well known to prevent dental caries by inhibiting enamel and dentin demineralization; hence, a high F concentration around crystals may possibly protect the crystals against dissolution^42^. A small F amount will on the other hand promote formation of fluoridated HAp^43^. In this study, F was clearly detected (Fig. 3). A high fluoride concentration was recorded in intratubular dentin and less in the intratubular precipitates. Fluoride was strongly found around a cluster of crystals in one specimen (Fig. 3B). Peritubular dentin is commonly mineralized up to a higher degree than intertubular dentin^44^; in intertubular dentin F can be detected in a higher amount^45^. Compared to the intratubular precipitates, peritubular and intratubular dentin are longer exposed to the oral environment, by which these tissues may absorb more F than the precipitates. In addition, the fluoride uptake in peritubular dentin may make it more resistant against caries demineralization as well as better protect the precipitates in the dentin tubules.

From our observations, we found that the dentin tubules in caries were extensively and deeply remineralized with precipitation of HAp and formation of amorphous calcium phosphate. The surrounding dentin was severely demineralized by the progressing caries. Especially 3D FIB-SEM imaging clearly disclosed intratubular precipitation, filling the dentin tubules with dense mineral deposits. This zone of filled dentin tubules inhibits bacterial penetration, thereby retarding caries progression. Several ions in the oral environment, such as Mg and F, may influence the process of nucleation and crystal growth during remineralization. To counteract caries as disease, clinically effective and biocompatible materials with remineralization capability should be developed^12^. A better understanding of the natural process of remineralization within carious dentin is essential, as it must be useful to develop bioactive materials that can effectively induce biomimetic remineralization.

## Methods

### Teeth preparation

Two extracted pre-molar teeth exhibiting deep dentinal caries from different persons were selected. Due to periodontal reasons or severe caries, dentists decided to extracted teeth with patients’ agreement. Informed consent was obtained from both participants according the guideline Ethical Guidelines for Medical and Health Research Involving Human Subjects. The protocol of this research was approved by the Commission for Medical Ethics of Okayama University Hospital, which in accordance with the guideline Ethical Guidelines for Medical and Health Research Involving Human Subjects (Approval number: No. 1606-020). Teeth were fixed using 2.5% glutaraldehyde (Sigma Aldrich, St. Louis, MO, USA), stained with 4% osmium tetroxide (Sigma Aldrich), gradually dehydrated using ascending ethanol concentrations and finally embedded in epoxy resin (Sigma Aldrich).

### SEM and FIB-SEM

**Figure 5:**
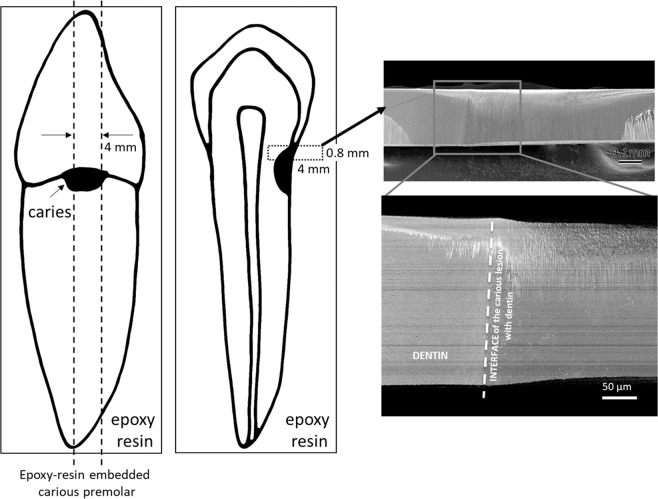
Schematic diagram explaining specimen preparation for FIB-SEM.

The teeth were cut into 0.8 × 4 × 4 mm squares including carious dentin using an auto cut-off machine (Accutom, Struers, Ballerup, Denmark) prior to having the cross-sections mechanically polished using diamond lapping films (3 M, St. Paul, MN, USA) and argon-ion polishing (SM-090101 Cross-Section Polisher, JEOL, Tokyo, Japan) (Fig. 5). Subsequently, a thin layer of carbon was coated on the surface (JEE-420T Vacuum Evaporator, JEOL), after which the specimens were examined using a field-emission-gun SEM (Feg-SEM; JSM-6701F, JEOL) operated at 5 kV using an annular semiconductor detector. Among the five carious teeth preliminary examined, two teeth revealed an area of remineralized dentin immediately adjacent of the caries lesion, which was further characterized using FIB-SEM and EDS-STEM.

Orthogonal FIB-SEM (SMF-1000, HITACHI High-Tech Science, Tokyo, Japan) imaging was conducted for 3D structural characterization. The FIB and SEM ion/electron beams were orthogonally aligned, instead of being set at the standard angle of ∼60°, for obtaining high-spatial resolution and high-contrast SEM photomicrographs^24^. Lucis Pro (Microtechnics, CA, USA) and Stacker and Visualizer-Kai software (System In Frontier, Tokyo, Japan) packages were utilized for 3D reconstruction. SEM observation was performed at an accelerating voltage of 0.5 kV. Images were taken by a mixture of two detectors; an annular in-lens secondary electron detector and an annular in-lens energy-selected backscattered electron detector. The observation area was 30 × 30 μm with 1000 pixels each. Serial-sectioning observations were carried out with a slice pitch of 30 nm for 1000 sheets, i.e. one voxel size is 30 nm.

### EDS-STEM and SAED

After serial sectioning by FIB-SEM, the successive plane of the remaining specimens was processed for EDS-STEM (Hitachi HD-2700, HITACHI High-Tech Science) operated at 200 kV and equipped with a silicon-drift type x-ray detector (AMETEK EDAX Octane 100 mm^2^, AMETEK EDAX, Mahwah, NJ, USA). TEM images and electron diffraction patterns were recorded with a JEM-2010F (JEOL) TEM operated at 200 kV.

## Supplementary information

Supplemental information.

Supplemental video.
