# Supplementary material for: Three-dimensional observation and analysis of remineralization in dentinal caries lesions
Source: Sci Rep. 2020 Mar 9;10:4387. doi: 10.1038/s41598-020-61111-1 (PMC7062784; doi:10.1038/s41598-020-61111-1)
Supplement: Supplementary file 1 — Supplemental information. [file 41598_2020_61111_MOESM1_ESM.docx]

**Three-dimensional observation and analysis of remineralization in dentinal caries lesions**

Kumiko YOSHIHARA^1,2,*^, Noriyuki NAGAOKA^3^, Akiko NAKAMURA^4^, Toru HARA^4^, Satoshi HAYAKAWA^5^, Yasuhiro YOSHIDA^6^, Bart VAN MEERBEEK^7^

^1^Okayama University Hospital, Center for Innovative Clinical Medicine, 2-5-1 Shikata-cho, Kita-ku, Okayama 700-8558, Japan; ^2^ National Institute of Advanced Industrial Science and Technology (AIST), Health Research Institute2217-14 Hayashi-cho, Takamatsu, Kagawa 761-0395 JAPAN; ^3^Okayama University Dental School, Advanced Research Center for Oral and Craniofacial Sciences, 2-5-1 Shikata-cho, Kita-ku, Okayama 700-8558 Japan; ^4^National Institute for Materials Science, 1-2-1 Sengen, Tsukuba, Ibaraki 305-0047, Japan; ^5^Okayama University, Graduate School of Interdisciplinary Science and Engineering in Health Systems, Biomaterials Laboratory, 3-1-1, Tsushimanaka, Kita-ku, Okayama 700-8530, Japan; ^6^Hokkaido University, Faculty of Dental Medicine, Department of Biomaterials and Bioengineering, Kita 13, Nishi 7, Kita-ku, Sapporo, Hokkaido, 060-8586 Japan; ^7^KU Leuven (University of Leuven), Department of Oral Health Research, BIOMAT & UZ Leuven (University Hospitals Leuven), Dentistry, Kapucijnenvoer 7, 3000 Leuven, Belgium

*Corresponding author: Kumiko YOSHIHARA [K-yoshi@md.okayama-u.ac.jp](mailto:K-yoshi@md.okayama-u.ac.jp)

**Figure legend for supplemental data:** 3D FIB-SEM reconstructions of the dentinal caries specimens shown in Fig. 2Aand 2B
